# Intraoperative Assessment of Margin Accuracy in Patients Diagnosed With Oral Squamous Cell Carcinoma in a Tertiary Hospital of Jharkhand: Frozen Section Versus Histopathology

**DOI:** 10.7759/cureus.58345

**Published:** 2024-04-15

**Authors:** Sunil Kumar Mahto, Aditi Kashyap, Manoj K Paswan, Saurav Banerjee, Satyabrata Patra

**Affiliations:** 1 Pathology, Rajendra Institute of Medical Sciences, Ranchi, IND

**Keywords:** accuracy, histopathological examination, margin assessment, intraoperative frozen section, oral squamous cell carcinoma

## Abstract

Background

The incidence of head and neck carcinoma is increasing. The use of an intraoperative frozen section plays a vital role in the evaluation of margin status in patients undergoing surgery for oral squamous cell carcinoma. A negative margin is not only an indication of successful surgery but also decreases the recurrence of disease and improves the overall survival of patients.

Aims and objective

The aim of this study is to assess the accuracy of margin in patients undergoing surgery for oral squamous cell carcinoma by intraoperative frozen section and compare it with conventional histopathological examination.

Methodology

The approach of our study was a hospital-based prospective study conducted on 28 patients diagnosed with oral squamous cell carcinoma. A frozen section was done on all patients undergoing surgery and compared with histopathological examination.

Results

Out of 28 patients undergoing surgery, the incidence of males was more than females, with a ratio of 6:1. The most common site of the tumor was left buccal mucosa comprising 28.57%, followed by gingivobuccal sulcus comprising 17.85%. In our study, the frozen assessed margin showed a sensitivity of 58.33%, specificity of 98.76%, and accuracy of 95.25%.

Conclusion

Frozen section is a reliable method for confirmation of margin accuracy and thus reduces the chance of re-surgery and recurrence of disease and increases overall patient survival.

## Introduction

Squamous cell carcinoma is the predominant type of oral cavity cancer, accounting for over 90% of cases. Intraoperative frozen section assessment plays a crucial role in managing these patients [1]. A successful cancer surgery is often indicated by a negative margin status confirmed through frozen section analysis. Despite the high accuracy of frozen section analysis in head and neck malignancies (reportedly over 99%), negative margins on frozen sections may not always translate to negative margins in the final pathology [2]. A positive resection margin can have several consequences for the patient. It has been linked to an increased risk of cancer recurrence, potentially impacting overall survival and influencing the course of future treatment decisions [3]. Consultation during surgery through the frozen section guides immediate surgical management and helps to establish the nature and type of lesion, confirming the presence of malignancy and the status of the margin involved or not. The margin accuracy is critical in the successful treatment of patients undergoing oral carcinoma surgery [4-6]. There are four studies conducted in the last 20 years that presented data helpful in accessing the concurrence rates between frozen section and permanent section diagnoses and the sensitivity and specificity of the procedure for the identification of squamous cell carcinoma at surgical margins [2,7-9]. Studies report an adequacy rate of around 97% for frozen section analysis, with a sensitivity of 83% and a specificity of 98% [7]. Discrepancies have been seen in the diagnosis between the frozen section and permanent section in tissue from the skin, breast, uterus, cervix, and thyroid [5-7].

While performing frozen section, errors of interpretation and errors of sampling can be seen, which include the initial selection of tissue by a surgeon, sampling of tissue by a pathologist, technical expertise required to prepare slides, errors in interpretation, and delivery of result back to surgeon [10-12].

The aim of this study is to assess the accuracy of margin in patients undergoing surgery for oral squamous cell carcinoma by intraoperative frozen section and compare it with conventional histopathological examination.

## Materials and methods

Study design and duration

A hospital-based, prospective, observation study was conducted at the Department of Pathology, Rajendra Institute of Medical Sciences from 2022 to 2023 after obtaining ethical approval (approval number: 95/IEC/RIMS).

Inclusion criteria

This study included all cases of biopsy-confirmed oral squamous cell carcinoma. The age of the patients over 15 years was taken in this study.

Methodology

All patients undergoing surgery were intraoperatively assessed with a frozen section. Margins were assessed clinically by a surgeon by tagging a long suture on the lateral margin and a short suture on the superior margin. The margin was inked for proper visualization. The tissue sample was secured in a chuck and then covered in a freezing medium, specifically designed for cryostat use. The chuck containing the tissue was placed in the cryostat chamber, which was pre-cooled and maintained between -20℃ and -25℃. At this temperature, the embedding media undergoes solidification. The tissue plane is adjusted and maintained parallel to the edge of the blade. After obtaining the appropriate section, the tissue was stained by rapid hematoxylin and eosin (H&E). The remaining tissue was fixed with formalin, put in paraffin wax blocks, sliced thinly, and then stained with H&E [13]. Both the slides from the three frozen sections and the permanent section were compared and any discrepancies were noted [13]. Any differences between the two were noted in the comments or microscopy section of the surgical pathology report.

Statistical analysis

Statistical analysis was done using SPSS software (IBM Corp., Armonk, NY). Categorical variables such as age, gender, and site of tumor were expressed with their mean. Assessment of three frozen sections for free margin status was done through sensitivity, specificity, positive predictive value, negative predictive value, and accuracy. Histopathological examination was taken as the gold standard test and results were compared.

## Results

Age and gender variation

In our study, we have taken 28 cases. The mean age of the patients was 57.35 years. The maximum age in this study was 78 years, and the minimum age was 30 years. Regarding the gender distribution, the male-to-female ratio was 6:1. The total percentage of male participants was 85.71% (24 cases out of 28) while the female participants were 14.29% (four cases out of 28) (Table 1).

**Table 1:** Age and gender variation M = male; F = female.

S. No. (N = 28)	Age	Gender
1	78	M
2	70	M
3	52	M
4	45	M
5	60	M
6	54	M
7	42	M
8	70	F
9	52	M
10	50	M
11	74	M
12	52	F
13	61	M
14	45	M
15	44	M
16	56	M
17	30	M
18	71	M
19	75	M
20	76	M
21	38	M
22	48	F
23	56	M
24	58	M
25	65	M
26	72	M
27	57	F
28	55	M

Site of lesion

In our study, the most common site of oral carcinoma was the left buccal mucosa, comprising 28.57% (eight out of 28), and the second most common site was gingivobuccal sulcus, comprising 17.85% (five out of 28) (Table 2).

**Table 2:** Location of oral squamous cell carcinoma

Diagnosis	Frequency (N = 28)	Percentage (%)
Carcinoma of left buccal mucosa	8	28.57
Carcinoma of right buccal mucosa	1	3.57
Carcinoma left lower alveolus	4	14.28
Carcinoma right lower alveolus	1	3.57
Carcinoma tongue	3	10.71
Carcinoma gingivobuccal mucosa	5	17.85
Carcinoma retromolar trigone	4	14.28
Carcinoma cheek	1	3.57
Carcinoma upper palate	1	3.57

Histological grade of the tumor

Different grades of tumor were analyzed in this study. Among these, the most common tumor was a moderately differentiated tumor, comprising 57.14% (16 cases out of 28) (Table 3).

**Table 3:** Grade of tumor

Grade of tumor	No. of cases (N = 28)	Percentage (%)
Well differentiated	9	32.14
Moderately differentiated	16	57.14
Poorly differentiated	3	10.71

Margin assessment

**Figure 1:**
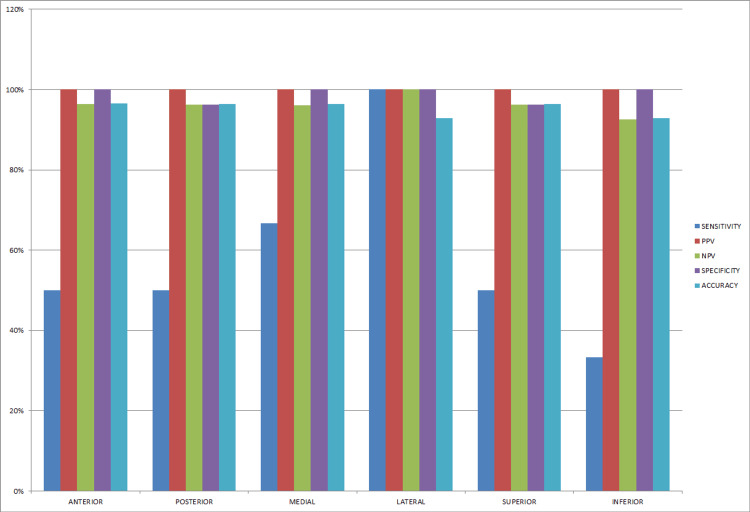
Sensitivity, specificity, and accuracy of intraoperative frozen section PPV = positive predictive values; NPV = negative predictive values.

Among 28 cases that underwent surgery, all cases were subjected to a frozen section. Different margins were analyzed in this study, including anterior, posterior, superior, inferior, medial, and lateral margins. The total number of the anterior margin was 28, the posterior margin was 28, the medial margin was 28, the lateral margin was 28, the superior margin was 28, and the inferior margin was 28. Margins subjected to frozen section were further confirmed by histopathological examination to see any discrepancy. We have considered paraffin-embedded histopathological examination as the gold standard test (Table 4 and Figure 1).

**Table 4:** Sensitivity, specificity, and accuracy of intraoperative frozen section PPV = positive predictive values; NPV = negative predictive values.

Margin	Sensitivity	PPV	NPV	Specificity	Accuracy
Anterior (N = 28)	50%	100%	96.42%	100%	96.55%
Posterior (N = 28)	50%	100%	96.29%	96.29%	96.42%
Medial (N = 28)	66.66%	100%	96.15%	100%	96.42%
Lateral (N = 28)	100%	100%	100%	100%	92.85%
Superior (N = 28)	50%	100%	96.29%	96.29%	96.42%
Inferior (N = 28)	33.33%	100%	92.59%	100%	92.85%

## Discussion

This study was conducted on 28 patients (N = 28). The mean age was 57.35 years and the male-to-female ratio was 6:1, comprising 85.71% (N = 24) males and 14.29% (N = 4) females. The increased incidence in the male population was due to the increased habit of tobacco chewing and poor oral hygiene. The most common site in this study was left buccal mucosa, followed by gingivobuccal sulcus, which was due to constant irritation of mucosa by betel and tobacco.

It is very important to achieve clear surgical margins in patients undergoing cancer surgery. While performing surgery, the aim of the surgeon is to achieve a negative microscopic surgical margin even if they have removed the tumor macroscopically. Frozen section is the most common technique that is used clinically to assess surgical margin [13].

Du et al. reported that 2% to 10% of frozen results, which were defined as negative based on intraoperative findings, could be positively detected in the postoperative pathology [2]. Whereas, in our study, 2.38% (four out of 168 frozen cases) of frozen results were negative, which detected positive on histopathology.

DiNardo et al. investigated the accuracy of frozen section analysis for margin assessment by examining 420 sections from 80 patients [7]. A 98.3% accuracy rate was found compared with permanent sections of the same tissue, regardless of the sub-site. They found a discrepancy rate of 1.6% (four false-positive and three false-negative margins), whereas, in our study, the accuracy rate for the anterior margin was 96.55%, the posterior margin was 96.42%, the medial margin was 96.33%, the lateral margin was 92.85%, the superior margin was 96.42%, and the inferior margin was 92.85%. Variations in results may be due to less sample size in our study.

In a cross-sectional study by Adhikari et al., frozen section analysis found 100% accuracy for margin assessment in all five oral cavity samples evaluated, supporting its potential role in oral cavity lesions [14]. In our study, 168 frozen sections were taken from different margins of oral squamous cell carcinoma, which were compared with the final histopathological report with an accuracy of 97.7% (164 cases out of 168).

We have taken the conventional histopathological examination test as the gold standard in our study, and the discrepancy in result was due to sampling error, inadequate sample, and folding of the tissue. Out of 168 frozen section samples, four samples show discrepancies when compared with histopathological examination. Overall, the frozen section has a sensitivity of 58.33%, specificity of 98.76%, and accuracy of 95.25%.

The limitation of this study was a smaller sample size for a clear comparison between the frozen assessed margin and the histopathologically assessed margin.

## Conclusions

The frozen section plays an important role in the intraoperative assessment of margin status in patients undergoing surgery for oral squamous cell carcinoma. Frozen section is a reliable method for confirmation of margin accuracy and thus reduces the chance of re-surgery and recurrence of disease and increases overall patient survival.
